# Supplementary material for: Effects of peppermint (Mentha piperita L.) oil in cardiometabolic outcomes in participants with pre and stage 1 hypertension: Protocol for a placebo randomized controlled trial
Source: PLoS One. 2025 May 7;20(5):e0321986. doi: 10.1371/journal.pone.0321986 (PMC12057884; doi:10.1371/journal.pone.0321986)
Supplement: Attachment — Submitted filename: REVIEWER COMMENTS.docx [file pone.0321986.s003.docx]

**REVIEWER 1**

**COMMENTS – Manuscript PONE-D-24-31138**

Title: “Effects of Peppermint (Mentha piperita L.) Oil in Cardiometabolic Outcomes in participants with pre and stage 1 hypertension: Protocol for a Placebo Randomized Controlled Trial.”

COMMENTS: Although there is no major flaw in the study/manuscript [and most of the things given below are already taken care of (except ‘Sample Size’ issue about I have serious concern), they are here because they are not described in the manuscript]. I have different opinion / observations/concerns or rather questions regarding a few issues which are given below:

Unfortunately, I see/found your ABSTRACT [though is well drafted (in my opinion)], is ‘assay type’. It is preferable [refer to item 1b of CONSORT checklist 2010: Structured summary of trial design, methods, results, and conclusions] to divide the ABSTRACT with small sections like ‘Objective(s)’, ‘Methods’, ‘Results’, ‘Conclusions’, etc. which is an accepted practice of most of the good/standard journals [including this one, though ‘The PLoS One Guidelines to Authors’ did not specify an Abstract format, it is desirable]. It will definitely be more informative then, I guess, whatever the article type may be {though Section headings may differ for different Article Types [example: Study Protocol]}.

**RESPONSE:** We agree with this, and also prefer the ‘structured’ abstract format. The previous trial protocols that we have read in PloS One were not in the structured format, so we believed that this is the format that the journal preferred. However, as you have indicated the journal guidelines do not specify a specific abstract format. We have now changed this section to a structured abstract in line with item 1b of the CONSORT checklist.

In my considered opinion, description given is section ‘Sample size’ (lines 144-150) is not adequate. In this context, I request authors to kindly note that according to table-2 on page 158 of Jacob Cohen’s paper “A power primer” in Psychological Bulletin, 1992, vol.:112, pp 155-159 [which is a sort of summary of the excellent book by Cohen himself titled ‘Statistical power analysis for the behavioral sciences’, Academic Press, 1977, New York] for medium effect size you need n=64 per group (type-I error=0.05, power=80%). You said “enabling a mean between group improvement in systolic blood pressure of 4.53 mmHg to be detected” that only 20 participants would suffice in each trial arm, i.e. a total N of 40. Is not the SD estimate required for estimation? It seems that you used very small SD [indirectly very large effect size] in estimation process. Please note that the ‘effect size’ assumed should have some basis (exact reference needs to be quoted) &/or reasonable/realistic, else the study is very likely ‘not to be able to’ detect a difference despite its presence}.

**RESPONSE:** We agree with you that we had not provided adequate information. We have now revised this section with more details. As there is really no data from a randomized trial in this population concerning peppermint, the sample size was based on our previous trial in healthy individuals. In this trial, we found a Cohen’s effect size of 0.81 for our primary outcome i.e. systolic blood pressure. As we would likely expect a larger effect in individuals with pre and state 1 hypertension, we pragmatically selected an effect size 0.85, which yielded a total N of 36 given the directionality of our hypotheses. Taking into account a 10% drop out rate which was pragmatically and conservatively selected based on our previous investigation in healthy individuals a total N of 40 was revealed.

It is appreciated that you said “This study adheres to the latest guidelines for reporting parallel-group randomized trials” (line 112) and quoted the reference [Moher, D., Hopewell, S., Schulz, K. F., Montori, V., Gøtzsche, P. C., Devereaux, P. J., & Altman, D. G. (2012). CONSORT 2010 explanation and elaboration: updated guidelines for reporting parallel group randomised trials. International journal of surgery, 10(1), 28-55] however, why the important term ‘CONSORT’ avoided/did not mention? {except in line 464 “Figure 2: Consort diagram describing the study design”} Although most of the important items are covered, few like ‘Allocation concealment’ (Item 9) are missing. Since your article type is ‘Clinical Trial’, you are supposed to cover these items in the report or even in ‘Protocol’ (even if you may not use them)]. Kindly make sure (confirm) that (line 117) the ‘Random Allocation Software’ proposed to be used, does allocation using randomly chosen ‘Permuted Blocks’ {as it may be known that ‘Permuted Block Randomization’ ensures same group sizes (not simple randomization) and that randomization is a process [not only sequence generation] which includes ‘Allocation Concealment’.

**RESPONSE:** We have now clarified allocation concealment within the manuscript and also clarified the nature of the random allocation – you are correct in that a Permuted Block Randomization will be adopted to meet the requirement for equal sized trial arms.

One question regarding ‘blinding’. Refer to lines 177-179: “in order to examine blinding efficacy, each participant will be asked to which trial arm that they felt that they had been allocated to at the conclusion of their post-intervention data collection session”, please clarify “How necessary it is to examine blinding efficacy”? In my opinion, sincere, meticulous execution of blinding principle/rule may/should suffice. Further, “Do you think the question you are proposed asking (each participant will be asked to which trial arm that they felt that they had been allocated to) will lead to correct/accurate answer pertains to achieved blinding efficacy”? Steps taken towards achieving perfect/ideal blinding [completely missing in the manuscript] are vital/important than finding achieved blinding efficacy, I guess.

**RESPONSE:** We do agree that it is vital to achieve an effective blinding strategy, however we feel that is it also very important to measure blinding efficacy in our study. There is a wealth of literature around blinding efficacy, which we will not seek to cite all of it here. However, a recent publication concluded that blinding reduces bias as preconceived notions (i.e. a lack of effective blinding) about the efficacy and safety of interventions, even subconsciously, may impact outcome expectancy and decisions about cointerventions. As such we strongly believe that it is necessary to examine the efficacy of our blinding strategy.

Yes, we are confident that the question that we ask participants in relation to blind. In placebo-controlled trials of supplements like that adopted in this trial, there is a sound foundation in adopting this technique within the literature.

We have now added additional detail regarding the blinding strategy to the manuscript.

Details of handling of control group and preparation of ‘placebo’ is very scarcely mentioned/described. Since this one (present article/manuscript) is an independent publication (not one in series), referring [lines 167-8: This method of placebo preparation has been shown by previous analyses to provide an effective blinding strategy] to old publication is not desirable. May not ‘duplicate’ but some details are required.

**RESPONSE:** We have now provided additional detail (as described above), in relation to the how we achieve an effective blinding strategy. There is no preparation per-se of the placebo solution itself, as this is simply the peppermint cordial.

I am sure that authors know the fact that questionnaires like Pittsburgh sleep quality index, Epworth Sleepiness Scale, Insomnia Severity Index, etc. [lines 229-230] are likely to yield data that are in [at the most] ‘ordinal’ level of measurement. In this context, I request authors to read a following note pasted from one famous standard textbook on ‘Medical Research Methodology’ :

Then application of suitable non-parametric (or distribution free) test(s) is/are indicated/advisable [even if distribution may be ‘Gaussian’ (also called ‘normal’)]. Agreed that there is/are no non-parametric test(s)/technique(s) available to be used as alternative in all situation(s), but should be used whenever/wherever they are available. Therefore, in short use suitable non-parametric test(s)/technique(s) while dealing with data that are in ‘ordinal’ level of measurement even if [despite that] the distribution may be ‘Gaussian’.

**RESPONSE:** This has now been added per your suggestion.

Because you stated in lines 268-270 that “To compare participant characteristics at baseline as well as compliance levels, between the trial arms, between-group linear mixed effects models will be utilized.”, please remember {pasted again from the same book, though I am sure that the authors already know these things}.

Statistical comparison of baseline characteristics when random allocation/assignment is used/done [often for good/standard/leading journals these days] is not required, because even if P-value(s) turn(s) out to be significant (while comparing baseline characteristics despite random allocation), it is, by definition, a false positive as you then are supposed to be testing ‘randomization’ then, which in any single trial may not balance all baseline characteristics (particularly when sample sizes are small).

**RESPONSE:** We agree with your recommendation and have now removed any reference to between group comparisons at baseline.

And also note that using linear mixed effects models is not wrong at all, but note that this technique [in fact any regression technique(s) for that matter] is/are not originally developed for testing the ‘Group difference(s)’. Head-to-head comparison is expected, as this is an indirect/secondary/by-product testing.

**RESPONSE:** We are satisfied that our adopted analyses of the trial primary and secondary outcomes is suitable to meet our study objectives.

I assume {because you stated in lines 281-2: that “The efficacy of blinding will be assessed using a one-way chi-square (Χ2) goodness-of-fit test”} that these learned authors know the limitations of Chi-square test [like more than 80% cells should have expected cell frequency more or equal to 5 as well as no cell frequency should be ‘zero’]. Though Chi-square test is very versatile and extremely useful, they are overlooked many times].

**RESPONSE:** We are aware of the potential limitations that you have expertly described, but we do not envisage that our specific analyses adopting this test will be subject to these drawbacks.

**REVIEWER 2**

Reviewer #2: Kindly rewrite the statement "whist these medicines are effective for the treatment of hypertensive disease, their long-term efficacy and cost-effectiveness have yet to be established " because as reference # 6 states the Long- term comparative effectiveness of antihypertensive monotherapies in primary prevention of cardiovascular events.

**RESPONSE:** We have now re-written.

Eradicate the grammatical mistakes and improve english.

**RESPONSE:** We have now done so.

Add a figure for the statistical analysis of paper.

**RESPONSE:** We do not believe that this would add anything to the manuscript and is not part of the CONSORT requirements.

Rationale and effects of papermint oil on sleep quality and other secondary outcomes.

**RESPONSE:** The rationale for sleep quality is already included within the questionnaire section as is examining psychological wellbeing. Sleep quality is diminished in those with cardiovascular and cardiometabolic disease as such, should peppermint be successful in improving blood pressure then there is a rationale for examining this outcome – this is already explained in the paper already
